# An open-access mobile compatible electronic patient register for rheumatic heart disease (‘eRegister’) based on the World Heart Federation’s framework for patient registers

**DOI:** 10.5830/CVJA-2015-058

**Published:** 2015

**Authors:** Joris van Dam, Brigitta Tadmor, Jonathan Spector, John Musuku, Liesl J Zühlke, Liesl J Zühlke, Mark E Engel, Bongani M Mayosi, Nick Nestle

**Affiliations:** Novartis Institutes for BioMedical Research, Cambridge, Massachusetts, USA; Novartis Institutes for BioMedical Research, Cambridge, Massachusetts, USA; Novartis Institutes for BioMedical Research, Cambridge, Massachusetts, USA; Department of Paediatrics & Child Health, University Teaching Hospital, Lusaka, Zambia; Western Cape Paediatric Cardiac Services, Red Cross War Memorial Children’s Hospital, University of Cape Town, Cape Town, South Africa; Department of Medicine, Groote Schuur Hospital and University of Cape Town, Cape Town, South Africa; Department of Medicine, Groote Schuur Hospital and University of Cape Town, Cape Town, South Africa; Department of Medicine, Groote Schuur Hospital and University of Cape Town, Cape Town, South Africa; Dimagi Inc, South Africa

**Keywords:** rheumatic heart disease, registries, mobile health, open-source model

## Abstract

**Background:**

Rheumatic heart disease (RHD) remains a major disease burden in low-resource settings globally. Patient registers have long been recognised to be an essential instrument in RHD control and elimination programmes, yet to date rely heavily on paper-based data collection and non-networked data-management systems, which limit their functionality.

**Objectives:**

To assess the feasibility and potential benefits of producing an electronic RHD patient register.

**Methods:**

We developed an eRegister based on the World Heart Federation’s framework for RHD patient registers using CommCare, an open-source, cloud-based software for health programmes that supports the development of customised data capture using mobile devices.

**Results:**

The resulting eRegistry application allows for simultaneous data collection and entry by field workers using mobile devices, and by providers using computer terminals in clinics and hospitals. Data are extracted from CommCare and are securely uploaded into a cloud-based database that matches the criteria established by the WHF framework. The application can easily be tailored to local needs by modifying existing variables or adding new ones. Compared with traditional paper-based data-collection systems, the eRegister reduces the risk of data error, synchronises in real-time, improves clinical operations and supports management of field team operations.

**Conclusions:**

The user-friendly eRegister is a low-cost, mobile, compatible platform for RHD treatment and prevention programmes based on materials sanctioned by the World Heart Federation. Readily adaptable to local needs, this paperless RHD patient register program presents many practical benefits.

## Background

Rheumatic heart disease (RHD) was largely eliminated from most high-income countries decades ago but it remains a major cause of cardiovascular disease in sub-Saharan Africa, indigenous Australia, south-central Asia, the Pacific region, and other low-resource settings globally.^1^,^2^ At least 15 to 20 million people are affected and more than 280 000 new cases are diagnosed each year,^3^ although recent data from populationbased screening using echocardiography suggest that the true prevalence could be up to tenfold higher.^4^–^6^

The World Heart Federation (WHF), an association of worldwide heart foundations and medical societies, is the leading international non-governmental organisation concerned with cardiovascular disease prevention.^7^ A key strategic target put forth by WHF is the use of comprehensive register-based control programmes in regions where RHD is endemic.^7^ Patient registers are instrumental in helping to organise the medical care of patients with RHD, minimising the loss to follow up, and maximising the likelihood of compliance with therapeutic regimens.^7^-^11^ This is particularly important in patients with RHD, many of whom require regular antibiotic therapy for decades in order to mitigate the progression of heart disease.

Registers also facilitate monitoring of longitudinal patient outcomes, and can be used to compile epidemiological data for use in programme planning and advocacy activities.^7^,^11^,^12^ Furthermore, patient registers can be used to collect, organise and report data required by national health authorities should RHD be considered a reportable disease. Examples such as the multi-national REMEDY study^13^ demonstrate the important roles that register data can play in research and in the design of effective RHD control programmes. A register variant can also be used to organise and store data in large-scale screening programmes to identify individuals with RHD who were previously undiagnosed. Until now, however, virtually all patient registers used in RHD programmes have relied on paperbased data collection and non-networked data-management systems, which limit their utility.

The emergence of mobile and cloud technologies, together with the increasing availability of low-cost mobile phones, computer tablets and data storage, offer the opportunity to explore the use of electronic patient registers in RHD control programmes in high-priority countries. We have developed such an electronic register tailored for specific use in a largescale comprehensive public–private effort to combat RHD in Zambia. This tool was demonstrated in February 2014, at the 2nd All Africa Workshop on Rheumatic Fever and Rheumatic Heart Disease in Livingstone, Zambia,^14^,^15^ and the delegates (representing 13 African countries) appealed for a version of the tool that could be incorporated into their own RHD programmes.^15^

To address this need, we sought to adopt the WHF’s framework for RHD patient register databases for an open-access mobile, compatible electronic platform (‘eRegister’) that would be user-friendly, modifiable to local contexts, inexpensive to operate, and straightforward to distribute. We then sought to assess the practical benefits of deploying such a system, and to make the eRegister freely available to potential users.

## Methods

## World Heart Federation’s RHD patient database tools

**Fig. 1.**
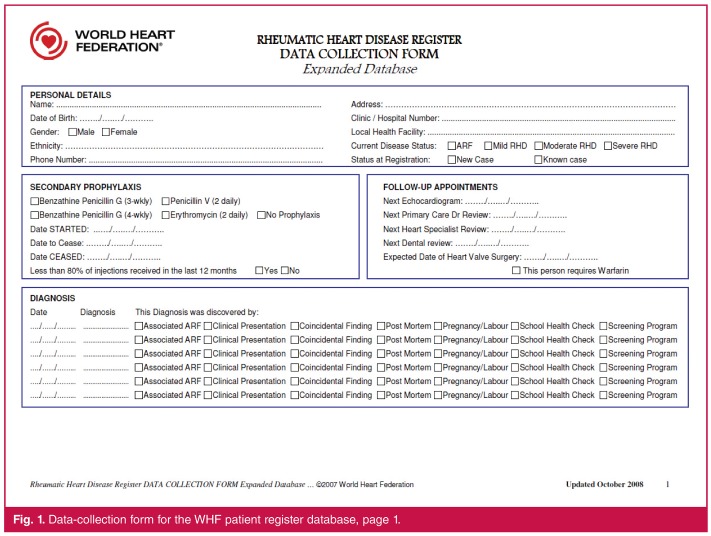
Data-collection form for the WHF patient register database, page 1.

**Fig. 2.**
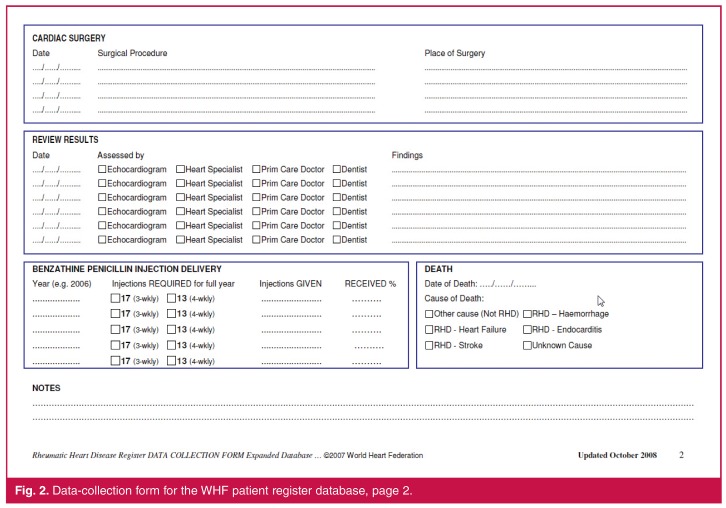
Data-collection form for the WHF patient register database, page 2.

The WHF has developed patient register database tools in support of RHD control programmes.^16^ Core components are downloadable from the WHF website and include (1) a datacollection form (Fig. 1, Fig. 2) meant to be printed and used by health workers to record a patient’s medical history, management plan and clinical outcomes; and (2) a complementary electronic Microsoft Access® database template that contains the same fields as the data-collection form. Using these tools, data would normally be entered by hand onto a printed data-collection form and then copied into the electronic database, which has inbuilt functionality to provide a rich variety of data reports, but in most cases would be non-networked and therefore accessible only from a single computer terminal.

## Electronic register system: development and testing

**Fig. 3.**
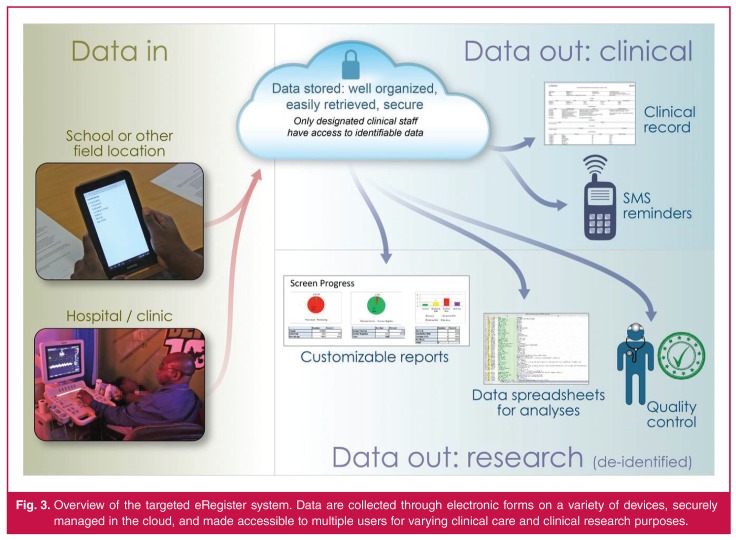
Overview of the targeted eRegister system. Data are collected through electronic forms on a variety of devices, securely managed in the cloud, and made accessible to multiple users for varying clinical care and clinical research purposes.

We aimed to replace the paper-based data-collection forms with electronic forms that could be deployed on inexpensive and widely available mobile devices (e.g. phones and tablets), and to associate these forms with a version of the WHF’s register database that would automatically and continuously be updated and readily accessible to multiple users simultaneously for patient care, data analysis and reporting purposes, as illustrated in Fig. 3.

We developed the electronic data-collection forms on the CommCare platform,^17^ an open-source, cloud-based software platform for health programmes that supports the development of customised data-capture tools using mobile devices. Our lead programmer (JVD) had general software programming experience only, with no prior expertise specific to CommCare. To become acquainted with the platform, the CommCare online training course was completed.^18^ We then developed a number of electronic data-collection forms by dividing the various sections of the WHF data-collection form into smaller sub-forms,^16^ as illustrated in Table 1. This approach provides greater flexibility for subsequently updating individual elements of the form and tailoring them to local needs.

**Table 1:** Overview of what forms are used to collect the data elements in the WHF patient register database and the eRegister. The data elements in the table refer to Figs 1 and 2.

	*WHF patient register database*	*eRegister*
*Data elements*	*Data collected on the following form*	*Data collected on the following form*
Personal details	Data-collection form	Data-collection form
Secondary prophylaxis	Data-collection form	Data-collection form
Follow-up appointments	Data-collection form	Schedule follow-up appointments
Diagnosis	Data-collection form	Diagnosis
Cardiac surgery	Data-collection form	Surgery
Review results	Data-collection form	Review results
Benzathine penicillin injection delivery	Data-collection form	Benzathine penicillin delivery
Death	Data-collection form	Death
Notes	Data-collection form	Data-collection form

Once the forms were developed, we created data-extraction jobs that export data from CommCare and map them to a spreadsheet in Microsoft Excel, with a table structure and data labels that are identical to the WHF database. We then created a copy of the WHF database in Microsoft Access® 2010 and used the standard data import features of the program to ‘link’ the database to the spreadsheet. Once established, subsequent data-extraction jobs can overwrite the spreadsheet with new data, which will automatically be visible in the WHF database.

We identified a number of minor technical issues for which we implemented adjustments in our copy of the WHF database, mainly to account for the different ways that data are represented in Microsoft Access® versus CommCare, such as the representation of missing values (‘ ’ or ‘–’), the representation of binary values (‘yes/no’ or ‘true/false’) and the use of date formats.

To pretest the resulting application, we used the sample data available in the WHF sample database.^16^ We entered the values from the sample database into our newly developed electronic data-collection forms, running on two different mobile devices: a general purpose seven-inch tablet running on the Android operating system (purchased commercially for US$59.99) and a general-purpose Android smartphone (purchased commercially for US$29.99). We extracted the data from CommCare into our copy of the WHF database, reviewed the data and the reports against the original WHF sample database, and confirmed their being identical.

## Field testing

A version of the eRegister was adapted to the specific needs of a school-based RHD screening programme in Lusaka, Zambia, in which health workers conducted clinical and echocardiographic assessment of schoolchildren in order to detect those with previously unrecognised RHD. Eight health professionals (including local nurses and radiographers, and programme management staff) were orientated on the use of the eRegister over two half-day training sessions and then received ongoing support as needed to utilise the tool.

The eRegister was deployed to support screening of 261 children in pilot screening sessions conducted from June to November 2014, and 1 022 children in full-scale screening that was conducted during February and March 2015. The mobile devices used were Samsung Galaxy Tab 2 tablets, which were sourced locally. Data were entered into the eRegister using a combination of tablets and laptops on site at schools and at the referral hospital.

## Results

The resulting eRegister application enables simultaneous data collection and entry. For example, field workers can directly enter patient data into the system’s electronic data-collection forms, which then automatically populate the cloud-based database, using either handheld mobile devices or computer terminals in clinics and hospitals. When data are entered on mobile devices, synchronisation with the central database takes place securely the next time a cellular or internet connection is established (i.e. wireless connection is not necessary at the time of data entry). Thus the database is continually updated, which streamlines its various clinical and research functions (Fig. 3).

The eRegister variables match those in the WHF register. Variables relating to existing data fields (e.g. names of villages or clinics) can easily be tailored to local needs and new data fields can be added. The system application itself can be installed to mobile devices by downloading from https://sites.google.com/site/rhderegister/home.

All access to the CommCare platform including mobile submissions is achieved through Hypertext Transfer Protocol Secure (HTTPS) and is cryptographically secure. Data stored in the eRegister is confidential and password-protected at all times.

We compared attributes of the eRegister and traditional paper-based systems. Benefits of the use of the eRegister system are likely to include:

Electronic data entry. Data are entered in electronic format directly at the point of capture, which obviates the need to manage paper forms, reduces the risk of data error associated with manual transcription of data from paper to electronic sources, and improves the ease of updating data.
Flexible, real-time system. Data collected through mobile devices populate the eRegister as soon as the device synchronises with the CommCare platform. Conversely, changes or local adaptions to data-collection forms to suit local needs can be made in real-time in the CommCare application and then distributed automatically to all mobile devices in the field. The next time the device is synchronised, the newly updated form will automatically be loaded onto the device, offering a level of flexibility in data collection that would be largely unworkable using paper forms.
Improving clinical operations. The eRegister system can be used to track and manage treatment of individual patients. Follow-up workflow plans can be created, penicillin allergies can be tracked and alternative prophylactic treatment can be prescribed, and custom reports and worklists can be created to help health workers manage a cohort of patients (for example, reports can be automatically generated that list patients who missed their last appointment). The system can also be utilised to distribute multimedia-format training modules to field workers.
Improving clinical outcomes. It is known that delivery of secondary prophylaxis within a registry-based programme increases the success of control programmes.^19^ The eRegister system can provide an integrated method to organise ongoing medical care of patients with RHD, minimising the loss to follow up, and maximising the likelihood of compliance with therapeutic regimens. This simple method also enables the monitoring of patient outcomes, and planning of advocacy and awareness activities in low-resource settings.
Improving treatment adherence. A particularly important example of improving clinical operations is the ease of implementing SMS reminders to the phones of patients, parents and health workers in advance of patient appointments, or in follow up to missed patient appointments. Adherence to prophylactic treatment for RHD has been shown to be low in many populations where RHD is endemic,^20^,^21^ and the use of register-based reminder systems can be an important tool to help support and improve adherence.^22^-^24^
Field team management. In addition to patient data, data relating to data-collection processes is automatically captured through the CommCare platform including, for example, the time taken to fill in an individual data-collection form or the number of forms submitted by each user. Data that describe patterns of data collection can be used to identify training needs and opportunities for productivity gains. Data collected by field teams can also be anonymised and reviewed by remote teams to analyse the quality of decision making and identify training needs. Multimedia training materials can be delivered to the field teams through the platform as well.
Research. A key feature of the system is the ability to rapidly generate de-identified data reports that can be used for research purposes. Furthermore, even though such data are currently not captured in the WHF register, the platform’s real-time data-collection features and workflow support can also be used to effectively support other processes critical to the conduct of clinical research studies, such as adverse event reporting.

Preliminary lessons learned from early field testing of the eRegister in Zambia include demonstration that the tool was overall easy to use, and that local programme staff were able to be trained to use the eRegister in a relatively short time and without specific prior technical knowledge or experience. The field team iteratively modified its work practices after the programme was underway in order to increase efficiency in its task of screening large numbers of children, for example, some data elements were collected in a different order and at a different location than was originally planned. It was straightforward to adapt the content and flow of forms in the eRegister to reflect changes in local work practices, and this was achieved in real-time without interruptions to the screening programme.

The study team reported several immediate benefits of the eRegister to programme operations. In particular, the eRegister’s actual and up-to-date status reports that could be generated at any time (including total number of children screened, where the screenings had taken place, how many children had screened positive for RHD, etc.), played important roles for programme monitoring and planning purposes. Another significant benefit was remote access to the eRegister by team members based in different locations, and functionality, which was applied to support data quality-assurance mechanisms.

There were also a number of challenges associated with the eRegister. Insufficient use was made of the available features to adapt the eRegister to evolving local work practices. Changes in work practices were not always reflected in corresponding changes in forms and workflows in the eRegister, leading to sub-optimal use of the tool. Poor internet connectivity at the sites in Zambia where the eRegister was used led to another intermittent problem; while the eRegister was always functional, it did on occasion take a long time to update software and upload large files to individual patient records (e.g. ultrasound images).

## Discussion

Rapid advances in technology over the past decade have made electronic patient resources theoretically within the reach of users in virtually every part of the world, including in low-resource settings where RHD is endemic and where efficient diseasecontrol programmes are most needed. We have adopted the WHF framework for patient register to develop an open-access, mobile, compatible, electronic patient register system. Our aim was not to attempt to develop a ‘one-size-fits-all’ RHD patient register, but rather to develop a platform that could be readily accessed by a wide range of stakeholders and adapted to their individual needs.

The main benefit of using an RHD register is to support longitudinal treatment programmes for patients diagnosed with RHD. In our field test of the eRegister in Zambia, we found that the tool could also be adapted to effectively support an RHD field screening programme. In addition to providing an efficient platform for managing data associated with screening programmes, the eRegister can foster compliance with the WHF guidelines for diagnosis of RHD25 by reproducing the criteria in the electronic forms, which are therefore readily accessible to health workers at the point of screening.

The platform is available at no cost, and provides countries and regions with an opportunity to adopt efficient, standardised patient register tools for the implementation of local RHD control programmes, to conduct RHD research studies, or to satisfy national reporting requirements should RHD be identified as a reportable disease. Moreover, further improvements can be made to the eRegister that perhaps would not be possible with the use of paper-based data-collection forms.

There are limitations of the eRegister system relative to traditional paper-based tools. Despite the fact that the ‘technology footprint’ with this system is relatively low, it will still be a barrier in some settings with regard to financial costs, familiarity by users, and need for ongoing troubleshooting and technical support. However, we believe these barriers can be effectively addressed by end-user training, and that the barriers will be lowered as the use of mobile technologies becomes more common among healthcare workers.

## Conclusions

The WHF register has been successfully converted to an openaccess eRegister platform. Preliminary results from a local RHD study in Zambia, for which a version of the eRegister was adapted, support the expected benefits of using an eRegister. Future improvements to the system (such as SMS reminders for families and integration with portable echocardiographic devices) can be added to the platform, as dictated by programmatic needs.
